# Development of Functional Ophthalmic Materials Using Natural Materials and Gold Nanoparticles

**DOI:** 10.3390/mi13091451

**Published:** 2022-09-01

**Authors:** Su-Mi Shin, Hye-In Park, A-Young Sung

**Affiliations:** Department of Optometry & Vision Science, Daegu Catholic University, Gyeongsan 38430, Korea

**Keywords:** natural substances, ginsenosides, gold nanoparticles, functional contact lenses, antibacterial properties

## Abstract

Ginsenoside, known as a natural substance, is a saponin component in ginseng and has various effects, such as antibacterial, antioxidant, and anti-inflammatory effects. In addition, gold nanoparticles can realize various optical and physical properties according to particle size and shape. For polymer polymerization, ginsenoside and gold nanoparticles were used as additives and copolymerized with a basic silicone hydrogel material. As gold nanoparticles, spherical and rod-shaped particles were used, and basic physical properties, such as water content, refractive index, and wettability of the prepared ophthalmic lenses, were measured. As a result of measuring the physical properties of the resulting polymer, it was found that the contact angle decreased by about 1.6% to 83.1% as the addition ratio of ginsenoside increased. In addition, as the addition ratio of metal nanoparticles increased, the refractive index was found to increase regardless of the shape of the nanoparticles. In addition, in the case of water content, the spherical shape gradually decreased according to the addition ratio, while the rod shape gradually increased according to the addition ratio. Therefore, it was found that the addition of ginsenoside, known as a saponin-based natural substance, has excellent wettability, and gold nanoparticles with different shapes have different properties. Thus, it is judged that the resulting copolymer can be utilized as a variety of highly functional ophthalmic polymer materials with high refractive index and high wettability.

## 1. Introduction

Hydrogel, one of the main materials constituting contact lenses, is mainly composed of 2-hydroxyethylmethacrylate (HEMA). In 1960, the first hydrophilic hydrogel lens was invented using HEMA as a material for soft contact lenses [1]. Since then, soft contact lenses have introduced to the public, and hydrogel lenses have been spotlighted as they have flexibility and high wetting property due to their hydrophilic properties, thereby reducing eye irritation. To compensate for the low oxygen permeability, which is the disadvantage of hydrogel lenses, silicone hydrogel has been introduced [2]. This material made by combining hydrogel with a silicone component with excellent oxygen permeability has increased oxygen permeability, but has a disadvantage in that water content and wetting property are degraded under the influence of silicone [3]. Since contact lenses, which are in direct contact with the cornea, have critical issues, such as their correlation with ophthalmic diseases and wearing comfort, many research studies are being undertaken on lens materials. In particular, the wearing comfort is greatly influenced by their properties, such as wetting property and oxygen permeability. In addition, natural materials with biocompatibility are widely used in various fields, and are also widely used as materials for ophthalmology by being incorporated into contact lens materials [4,5]. In particular, ginsenoside, a saponin-based natural substance, is an active compound that supports the assertion of the efficacy of ginseng, exhibiting antioxidant, antibacterial, anticancer, and immune activity [6,7,8]. Furthermore, with its bipolar property, it has excellent compatibility in various materials. In particular, the ginsenoside Rg3, one of saponin components, has been reported to have an anti-inflammatory effect on dry eye syndrome [9,10]. In that context, this study was intended to obtain a positive effect by incorporating ginsenoside, a natural substance, into contact lens materials. Recently, due to the COVID-19 pandemic, interests are focused on antibacterial activity around the world, and accordingly, the industry related to the materials of medical devices is spurring the development of antibacterial materials [11,12]. In the case of contact lenses, products with functionality, such as antibacterial activity, are leading the market [13,14]. In particular, even with a small amount of gold nanoparticles added, they have excellent antibacterial properties, and therefore, they have been spotlighted in various fields [15]. In addition, gold nanoparticles have various properties according to the size and type of the particles and can implement various colors and functions according to the wavelength range [16]. Since antibacterial properties can reduce the side effects of contact lenses, their utility is great, and the functionality of hydrogel contact lenses can be improved by utilizing various properties of gold nanoparticles. In that context, in this study of hydrogel contact lens materials that can reduce ophthalmic side effects by improving the functionality of existing hydrogel lenses, the possibility of their application as various ophthalmic lens materials was confirmed by examining the properties of ginsenoside and gold nanoparticles used as additives.

## 2. Experimental Details

### 2.1. Reagents and Materials

**Figure 1:**
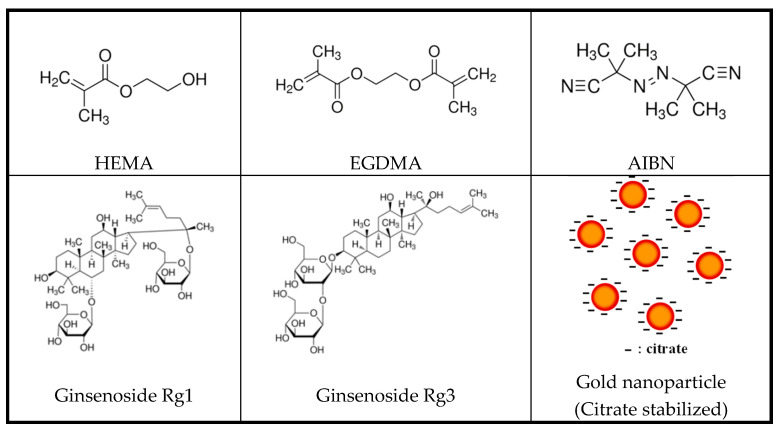
Chemical structures of monomers and additives.

Interventionary studies involving animals or humans and other studies that require ethical approval must list the authority that provided approval and the corresponding ethical approval code. In this study, 2-hydroxyethylmethacrylate (HEMA, Sigma-Aldrich, St. Louis, MO, USA), one of the main materials of the hydrogel lens; ethylene glycol dimethacrylate (EGDMA, Sigma-Aldrich, St. Louis, MO, USA), a crosslinking agent; and azobisisobutyronitrile (AIBN, Sigma-Aldrich, St. Louis, MO, USA), a thermal initiator, were copolymerized. In addition, two types of natural ginsenosides, Rg1 and Rg3 (Sigma-Aldrich, Ruelzheim, Germany), were used. In addition, gold nanoparticles were used as additives, and sphere-shaped gold nanoparticles and rod-shaped nanoparticles with an aspect ratio of 3 times or more were selected. The nanoparticles were purchased from Fine Nano Co., Ltd. (253, Samseong-ro, Yeongtong-gu, Suwon-si, Gyeonggi-do, Seoul, South Korea). For all other reagents, Sigma-Aldrich products were used. The structural formulas of the materials used in the experiment are presented in Figure 1.

### 2.2. Reagents and Materials

A basic combination was formed using HEMA, EGDMA, and AIBN, and the ginsenosides Rg1 and Rg3 were added at a ratio of 0.1% to 2%, respectively, to prepare a hydrogel contact lens. In addition, a new basic combination was formed by selecting the type of ginsenoside and various ratios, and then, gold nanoparticles were added at a ratio of 0.1% and 0.2%, respectively, to prepare hydrogel lens. In addition, the sample containing the gold nanoparticles was uniformly dispersed for about 1 h using an ultrasonic disperser (Branson 2510, Branson, Mexico) for dispersion, and each monomer was stirred using a vortex. All contact lenses were thermally polymerized at 100 °C for 1 h using a cast molding method. The samples prepared by the basic combination were named Ref and were named 1-A, 1-B, and 1-C and 3-A, 3-B, and 3-C, according to the type and amount of ginsenoside added. In addition, 3-C was named G-Ref and named GS-1, GS-2, GR-1, and GR-2 according to the type and amount of gold nanoparticles added to improve functionality. The mixing ratios of the prepared lenses are presented in Table 1.

### 2.3. Instruments and Analysis

Each of the prepared lenses was hydrated in 0.9% sodium chloride (Dai Han Pharm Co., Ltd., Seoul, Korea) physiological saline for 24 h, and optical/physical properties, such as spectral transmittance, refractive index, water content, contact angle, and water content, were evaluated. Spectral transmittance was measured based on ISO 8599:1994, and chromaticity was measured using a colorimeter capable of measuring transmittance and color difference, and the chromaticity of each sample was analyzed through coordinates. In addition, the refractive index and water content were measured based on ISO 18369-4:2006, and the water content was measured using a gravimetric method. The tensile strength was evaluated by measuring the highest value at which the lens breaks at the time a force of 0 to 2.00 kgf is applied to both sides of the sample at a rate of 10 mm/1 min. In addition, the surface structure and roughness of the prepared lens samples were analyzed using the contact angle, AFM and SEM, and the contact angle was measured using the sessile drop method. For antibacterial properties, antibacterial properties against Staphylococcus aureus were analyzed using 3M Petrifilm^TM^ (Sigma-Aldrich, St. Paul, USA) a dry film medium, and the extracts were evaluated by KMnO_4_ reduction test and absorbance measurement. All the experimental values in this study were repeatedly measured 5 times, and the average values were presented.

## 3. Results and Discussion

### 3.1. Hydrogel Lenses Containing Ginsenoside Materials

The optical and physical properties and surface properties were analyzed according to the addition ratio of the ginsenoside Rg1 and Rg3 added in the hydrogel lens, respectively.

#### 3.1.1. Optical Properties

**Figure 2:**
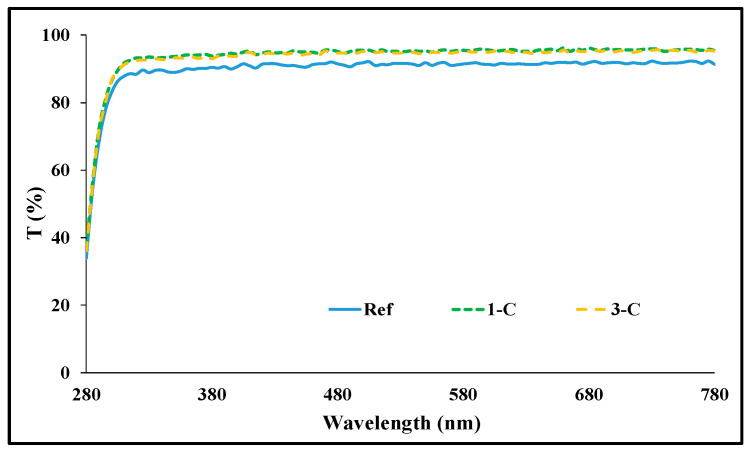
Spectral transmittances of samples (Ref, 1-C, 3-C).

Spectral transmittance was measured by classifying it into UV-B (280–315 nm), UV-A (315–380 nm), and visible light region (380–780 nm). As a result of the measurement, in the Ref prepared in the basic combination, it was 72.35%, 89.56%, and 91.47%. In the case of Rg1 combination with ginsenoside added, it was 74.10–75.60%, 91.03–93.71%, and 92.66–95.37%; and in the case of the Rg3 combination, it was 72.50–75.22%, 90.21–93.02%, and 91.98–94.90%, depending on the amount added. In conclusion, it was confirmed that with the addition of ginsenoside, the transmittance increased, allowing for the preparation of an optically excellent lens. A light transmittance graph of each sample is presented in Figure 2, respectively.

#### 3.1.2. Physical Properties

As a result of measuring the refractive index and water content of Ref, it was 1.4355% and 38.44%, respectively. However, in the case of the Rg1 combination, the change in the physical properties of the water content and refractive index was insignificant in comparison with Ref. On the other hand, in the case of the Rg3 combination, the water content increased as the amount of addition increased, and the refractive index was inversely proportional to the water content. The physical properties of the prepared lenses are presented in Table 2.

**Figure 3:**
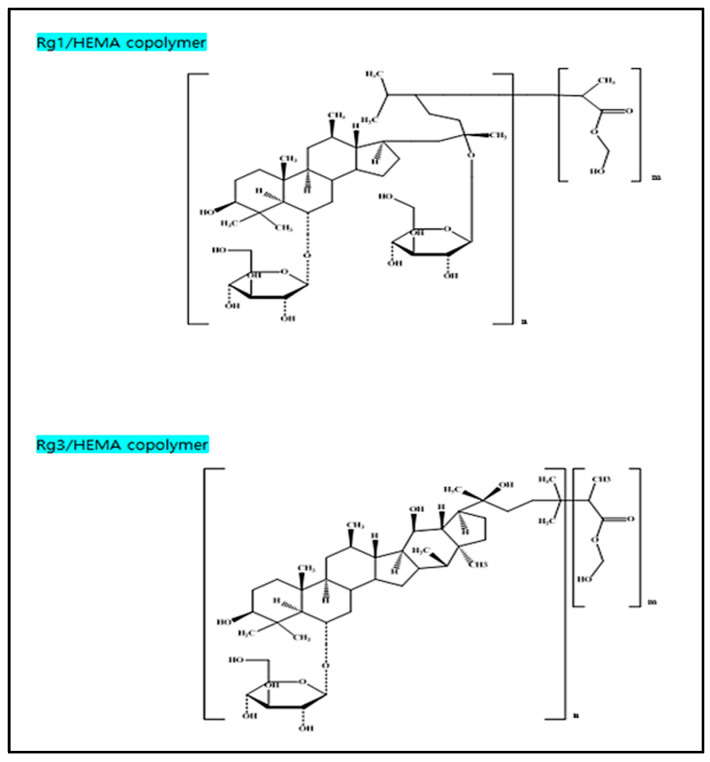
Produced Rg1/HEMA copolymer and Rg3/HEMA copolymer.

The produced polymer for each of rg1 and rg3 is presented in Figure 3, and the hydrophilicity of each monomer could be confirmed by measuring the water content and wettability of the produced lens polymer. Based on this, the samples containing gold nanoparticles were prepared, and the characteristics were confirmed.

#### 3.1.3. Surface Analysis

**Figure 4:**
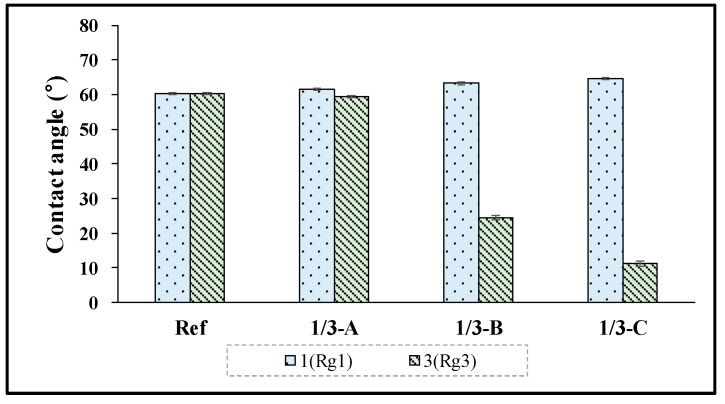
Contact angles of samples (Ref, 1/3-A, 1/3-B, 1,3-C).

To analyze the surface properties of hydrogel contact lenses with ginsenoside added, the contact angle was measured. As a result, it was found that in Ref, it was 60.26°, and in the Rg1 combination, it was 61.50°–64.50°, indicating a slight increase in the contact angle compared with Ref. On the other hand, in the case of the combination of Rg3, in 3-A, it was 59.30°; in 3-B, it was 24.40°; and in 3-C, it was 11.31°, indicating that the wetting property was significantly improved as the contact angle decreased with the increased addition ratio of Rg3. The measurement results are presented in Figure 4.

**Figure 5:**
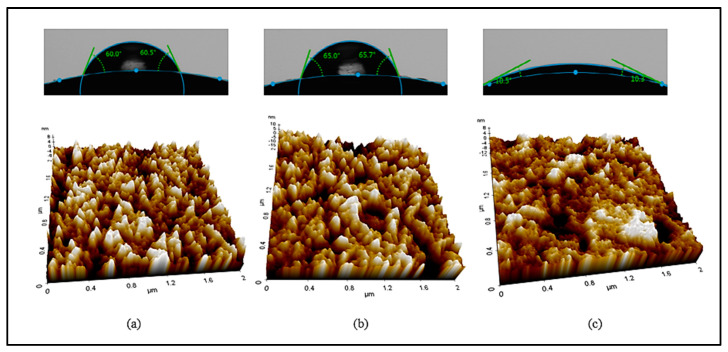
Contact angles and AFM images of samples ((**a**) Ref, (**b**) 1-C, (**c**) 3-C).

In addition, AFM analysis was used to examine the lens surface, and the average values of the surface roughness of the Ref, 1-C, and 3-C samples were compared. As a result, the average surface roughness value (Ra) of Ref was 2.1 nm, that of 1-C was 3.4 nm, and that of 3-C was 1.7 nm. The resultant values of the wetting property and surface roughness were proportional, and the surface of the 3-C group with the best wetting property was found to be the smoothest. In that context, the physical properties of the hydrogel lens varied according to the addition of the ginsenosides Rg1 and Rg3; and, in particular, the addition of Rg3 was found to improve the wetting property, water content, and visible light transmittance of the lens. The contact angle and AFM measurement images for the ginsenoside Rg3 are presented in Figure 5.

### 3.2. Hydrogel Lenses Containing Gold Nanoparticles

A new basic combination was formed by selecting HEMA, EGDMA, AIBN, and 2% ginsenoside Rg3. Gold nanoparticles in different shapes were added to the basic combination at ratios of 0.1% and 0.2%, and the functionality of the prepared contact lenses was evaluated.

#### 3.2.1. Optical Properties

**Figure 6:**
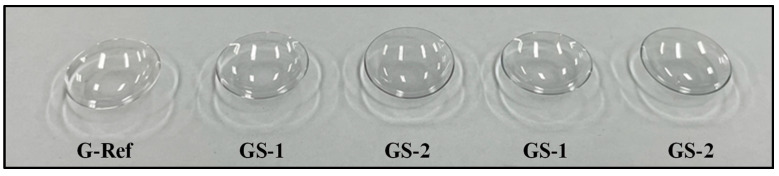
Produced hydrogel lens appearance of samples.

The optical properties of hydrogel lenses with sphere-shaped and rod-shaped gold nanoparticles added were evaluated. The lenses prepared by adding gold nanoparticles exhibited a blue color as a whole in both sphere-shaped and rod-shaped lenses. The images of the prepared lens are presented Figure 6.

**Figure 7:**
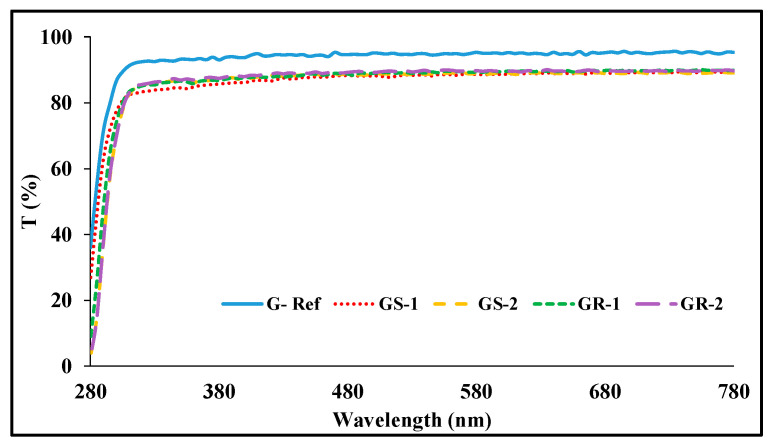
Spectral transmittance of samples containing gold nanoparticles.

With regard to the spectral transmittance of the sphere-shaped gold nanoparticles, GS-1 exhibited transmittances of 63.97%, 84.45%, and 88.45%, and GS-2 exhibited transmittances of 54.92%, 86.09%, and 89.22%. In addition, in the case of the spectral transmittance of the rod-shaped gold nanoparticles, GR-1 exhibited transmittances of 49.13%, 86.80%, and 89.46%, respectively, and GR-2 exhibited transmittances of 48.87%, 86.35%, and 88.75%. Under the influence of the gold nanoparticles, the transmittance decreased again compared with that of G-Ref, but it was found that the UV-B region was blocked to some extent by the addition of the gold nanoparticles regardless of the type of nanoparticles. In particular, the sample with rod-shaped gold nano added exhibited a higher blocking rate to some extent compared with the sphere-shaped sample. The measurement results of light transmittance are presented Figure 7.

**Figure 8:**
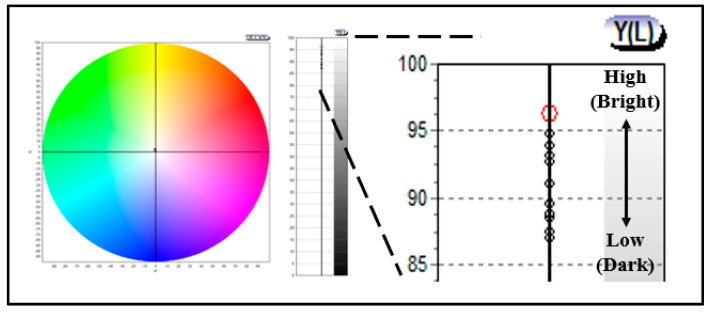
Color values of samples containing gold nanoparticles.

In addition, to evaluate the chromaticity of the prepared lens, it was analyzed using a colorimeter, and color coordinates in CIE and Y(L) using R (700 NM), G (546 nm), and B (435 nm) were confirmed. As a result of the measurement, it was found that there was no significant difference in the CIE color coordinates, and in Y(L), which is the brightness, it exhibited a value of 96.21–87.42, indicating that the brightness decreased as the gold nano addition ratio increased. In particular, it was found that the brightness color coordinates of the lens with rod-shaped gold nanoparticles added were lower to some extent. The numerical value L (Lightness) representing the brightness exhibited white light close to 100, and exhibited a black color close to 0. In that context, as the amount of gold nanoparticles added increased, the color of the prepared lens gradually became darker, and thus, it was confirmed that the brightness was lowered. In addition, as the chromaticity increased, the spectral transmittance seemed to have decreased slightly, and it exhibited a value of visible light transmittance of 87% or more, indicating the possibility of its optical application. The chromaticity measurement results for each sample are presented in Figure 8.

#### 3.2.2. Physical Properties

**Figure 9:**
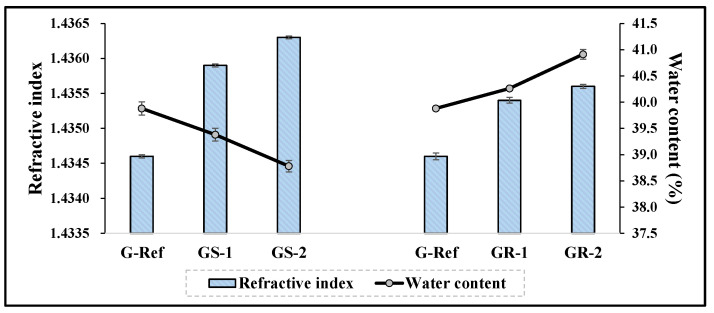
Refractive index and water content of hydrogel lens samples containing gold nanoparticles.

**Figure 10:**
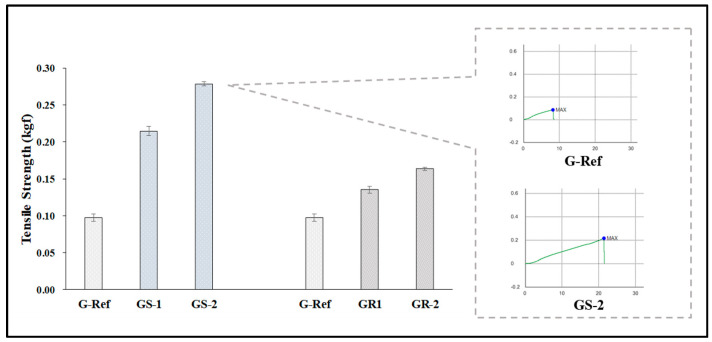
Tensile strength of samples containing gold nanoparticles.

To examine the physical properties according to the addition of nanoparticles, the refractive index, water content, tensile strength, and oxygen permeability of the prepared lens were measured. As a result of comparing them with those of G-Ref, the refractive index and tensile strength gradually increased according to the amount of gold nanoparticles added regardless of the type of gold nanoparticles, and in particular, the refractive index and tensile strength of the lenses with sphere-shaped gold nanoparticles added were high, which was considered to be influenced by the refractive index, and the tensile strength increased in all samples with gold nanoparticles added. In addition, in the case of water content, it was found that the water content of the samples with the sphere-shaped gold nanoparticle decreased according to the ratio, whereas the water content of the samples with the rod-shaped nanoparticle increased. Additionally, as a result of measuring the oxygen permeability, all samples were about 11.45–12.04 × 10^−11^ (cm^2^/s) (mlO_2_/mL × mmHg), which was slightly higher than that of a typical hydrogel contact lens sample. The water content and refractive index results are represented in Figure 9, and the tensile strength results are represented in Figure 10.

#### 3.2.3. Surface Analysis

**Figure 11:**
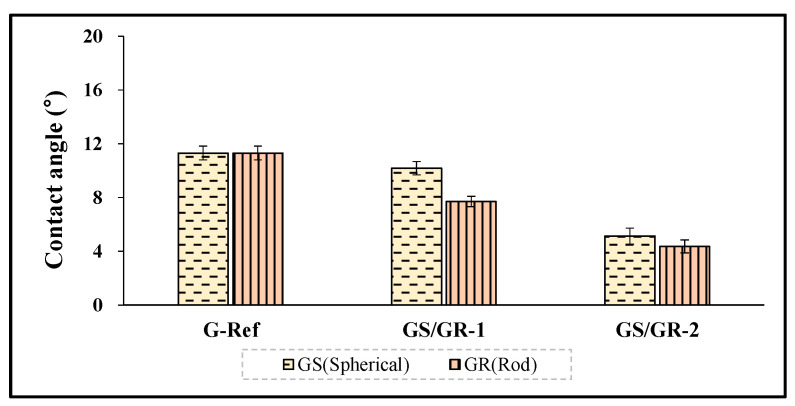
Contact angle of samples containing gold nanoparticles.

To examine the wetting property of the hydrogel lens with gold nanoparticles added, the contact angle was evaluated, and to examine the surface roughness and surface condition according to the presence or absence of furnace particles, AFM and SEM analysis methods were used, respectively. The contact angles of Ref and G-Ref were 60.26° and 11.31°, respectively, and in the case of the groups with ginsenoside and gold nanoparticles added, GS-1 was 10.18°, GS-2 was 5.13°, GR-1 was 7.70°, and GR-2 was 4.36°. In that context, it was confirmed that the wetting property was improved with the addition of gold nanoparticles, and in particular, the rod-shaped particles further improved the wetting property of the lens. The contact angle results of each sample are presented in Figure 11.

**Figure 12:**
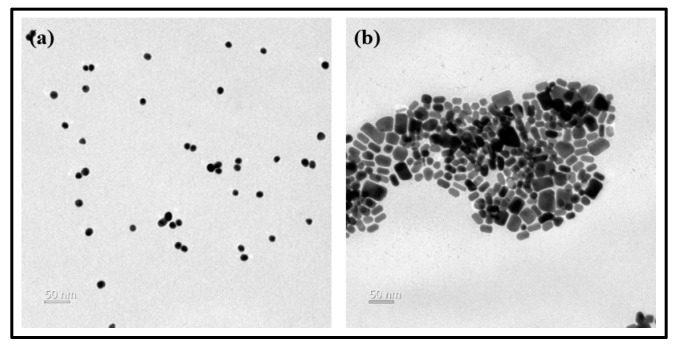
Gold nanoparticle analysis by TEM images ((**a**) sphere-shaped, (**b**) rod-shaped).

**Figure 13:**
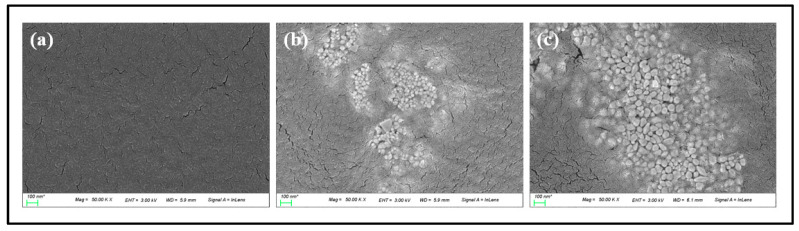
Surface analysis by SEM images ((**a**) G-Ref, (**b**) GS-2, (**c**) GR-2).

To examine the surface condition of the lens according to the change of the contact angle, AFM was used, and the average surface roughness for G-Ref, GS-2, and GR-2 was measured. As a result, the average surface roughness was 1.7 nm for G-Ref, 2.6 nm for GS-2, and 2.7 nm for GR-2, indicating that the surface roughness of the lens with gold nanoparticles added increased. In that context, the change in wetting property due to the addition of gold nanoparticles did not suggest a significant correlation with the surface roughness. Although the surface roughness slightly increased due to the influence of sphere-shaped and rod-shaped gold nanoparticles, the generation of a synergistic effect with gold nanoparticles and ginsenosides is considered to lead to a significant improvement in the wetting property. In addition, as a result of examining the shape of the gold nanoparticles used through TEM, it was confirmed that the nanoparticles had spherical and rod-shaped particles and various sizes ranging from 10 to 100 nm. As a result of examining the presence or absence of sphere-shaped and rod-shaped gold nanoparticles in the contact lens material through SEM, it was confirmed that the nanoparticles with sizes of about 15–30 nm for GS-2 and about 50–100 nm for GR-2 were distributed on the lens surface. TEM and SEM analysis images for each sample are presented in Figure 12 and Figure 13, respectively. 

#### 3.2.4. Stability

**Figure 14:**
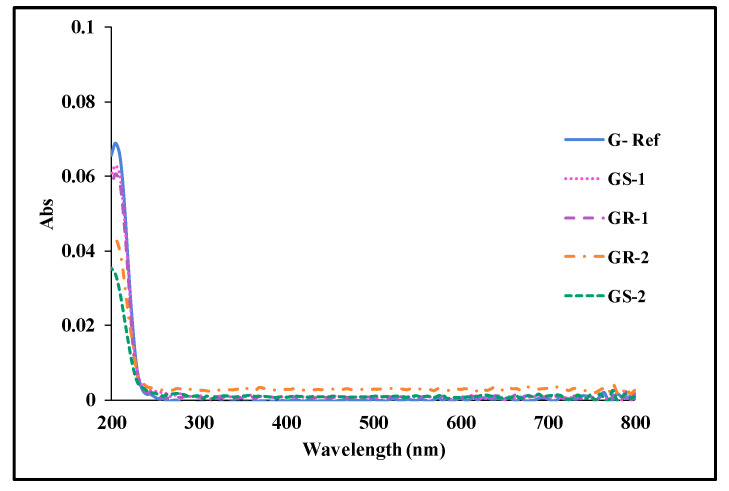
Absorbance of samples containing gold nanoparticles.

To evaluate the polymerization stability according to the addition of gold nanoparticles, the eluate was examined using the KMnO_4_ reduction test and the absorbance measurement. The prepared lens was heated to 70 °C for 24 h and then measured. In the potassium permanganate reduction test, the control group was set to distilled water, and the experimental group was set to G-Ref, GS-2, and GR-2. As a result of the potassium permanganate reduction test, the difference in eluate values in all groups was measured to be less than 2.0 mL, exhibiting excellent polymerization stability regardless of the presence or absence of gold nanoparticles. In addition, as a result of measuring absorbance, light was absorbed the most at 205 nm, and all groups showed an absorbance of less than 0.10, exhibiting excellent polymerization stability. It was also confirmed that the addition of gold nanoparticles leads to a smaller amount of eluate compared with G-Ref, thereby improving the polymerization stability. The eluate results for each sample are summarized in Table 3, and the absorbance graphs are presented in Figure 14.

#### 3.2.5. Antibacterial Property

**Figure 15:**
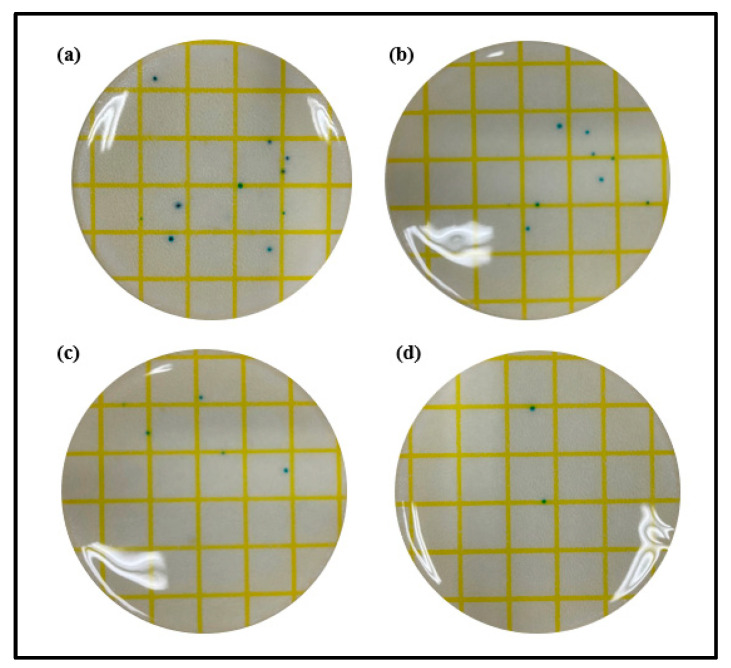
Antimicrobial property of samples ((**a**) Ref, (**b**) G-Ref, (**c**) GS-2, (**d**) GR-2).

Staphylococcus, the main cause of ocular infections caused by microorganisms, is one of the most common strains that cause bacterial keratitis and bacterial corneal ulcers. In that context, to evaluate the antibacterial properties of the lenses prepared according to the presence or absence of ginsenosides and gold nanoparticles, the antibacterial properties against Staphylococcus aureus were analyzed using 3M Petrifilm^TM^, a dry film medium. As a result of measuring the antibacterial properties of Ref, a rather large number of microorganisms were confirmed in the staphylococcal medium. Additionally, in G-Ref with the ginsenoside Rg3 added, the microbial count slightly decreased compared with Ref, but there was no significant difference. On the other hand, GS-2 and GR-2 exhibited superior antibacterial properties compared with Ref and G-Ref. In particular, the GR-2 group, rod-shaped gold nanoparticles, exhibited excellent antibacterial properties against Staphylococcus aureus. The results of measuring antibacterial properties for each sample are presented in Figure 15.

## 4. Conclusions

The hydrogel lens material prepared in this study is a functional material intended to improve the wearing comfort. The natural substances Rg1 and Rg3 were used in the basic combination, and the sphere-shaped and rod-shaped gold nanoparticles with different shapes were used as additives for the further improvement of functionality. In the case of the sample containing ginsenoside, the wetting property of Rg3 was very high compared with Rg1, and the water content and visible light transmittance were improved in addition to the wetting property, indicating that the water content and visible light transmittance were improved, thereby improving optical excellence and, at the same time, improving the functionality that determines wearing comfort. In addition, in the case of gold nanoparticles, it was confirmed that UV protection properties, antibacterial properties, and wetting property were excellent, and polymerization stability according to the addition of gold nanoparticles was also improved. It is thus considered that if the ginsenoside Rg3 and gold nanoparticles (sphere-shaped and rod-shaped) are properly used in an appropriate manner, lenses with a wide range of functionalities, such as excellent wetting property, antibacterial properties, and tensile strength, can be manufactured, thereby compensating for the problems of existing hydrogels used in ophthalmology.

## Figures and Tables

**Table 1:** Percent compositions of samples (Unit: wt%).

	HEMA	EGDMA	AIBN	Ginsenoside Rg1	Ginsenoside Rg3	Spherical Gold NPs	Rod Gold NPs
Ref	99.30	0.50	0.20	-	-	-	-
1-A	99.21	0.50	0.20	0.10	-	-	-
1-B	98.81	0.49	0.20	0.50	-	-	-
1-C	97.36	0.49	0.19	1.96	-	-	-
3-A	99.21	0.50	0.20	-	0.10	-	-
3-B	98.81	0.49	0.20	-	0.50	-	-
3-C (G-Ref)	97.36	0.49	0.19	-	1.96	-	-
GS-1	97.26	0.49	0.19	-	1.96	0.10	-
GS-2	97.16	0.49	0.19	-	1.96	0.20	-
GR-1	97.26	0.49	0.19	-	1.96	-	0.10
GR-2	97.16	0.49	0.19	-	1.96	-	0.20

**Table 2:** Physical properties of contact lens samples.

	Ref	1-A	1-B	1-C	3-A	3-B	3-C
Refractive index	1.4355	1.4353	1.4354	1.4355	1.4353	1.4349	1.4346
Water content (%)	38.58	38.66	38.60	38.65	38.86	39.43	39.88

**Table 3:** Extractable leachable testing of samples.

	KMnO_4_ Reduction Test (mL)	Absorbance Measurement
Distilled water	20.57	-
G-Ref	20.22	0.069
GS-2	20.17	0.035
GR-2	20.01	0.043

## Data Availability

Not applicable.
